# Editorial: Early Origins of Psoriatic Arthritis

**DOI:** 10.3389/fmed.2021.794229

**Published:** 2021-11-08

**Authors:** David Simon, Abdulla Watad, Santiago Rodrigues-Manica, Carlo Perricone

**Affiliations:** ^1^Department of Internal Medicine 3, Friedrich-Alexander University (FAU) Erlangen-Nuremberg and Universitätsklinikum Erlangen, Erlangen, Germany; ^2^Deutsches Zentrum fuer Immuntherapie (DZI), FAU Erlangen-Nuremberg and Universitätsklinikum Erlangen, Erlangen, Germany; ^3^Rheumatology Unit, Department of Medicine B, Zabludowicz Center for Autoimmune Diseases, Sheba Medical Center, Ramat Gan, Israel; ^4^Sackler Faculty of Medicine, Tel Aviv University, Tel Aviv, Israel; ^5^Leeds Institute of Rheumatic and Musculoskeletal Medicine (LIRMM), University of Leeds, Leeds, United Kingdom; ^6^Department of Rheumatology, Universidade NOVA de Lisboa, Lisbon, Portugal; ^7^Rheumatology Unit, Department of Medicine, University of Perugia, Perugia, Italy

**Keywords:** psoriatic arthritis, transition, risk factors, Psoriasis, intervention, enthesitis

Psoriasis (PSO) is a chronic inflammatory skin disease affecting 1–3% of the general population. An estimate of 10–30% of PSO patients develop psoriatic arthritis (PsA) with arthritis, dactylitis, enthesitis, and axial involvement during their lifetime. PsA is associated with structural damage, reduced quality of life, disability, and mortality (1). Clinically apparent PsA is often associated with systemic inflammation, structural damage of the joints may be noted, and eventually resulting in physical functioning limitation. Thus, early detection of PsA can lead to better outcomes and prevent irreversible damage. A deeper understanding of the early phase of the disease is important to achieve better outcomes, avert damage, and allow better, and more detailed risk stratification of PSO patients at-risk for disease progression. It is known that particular clinical [for example, presence of nail involvement of PSO patients (2)], serological [e.g., CXCL10 (3)], or imaging factors [subclinical inflammation and osteoproliferative changes at entheseal areas (4, 5)] have a good prognostic value. Despite these important findings, further knowledge about the early phase of PsA and the in-depth characterization of patients at-risk for developing PsA is needed. Basically, the example of the PSO-PsA continuum illustrates the importance of new concepts, especially molecular classification concepts, to fully capture different aspects of inflammation in psoriatic disease (6).

In our Research Topic, several reviews (Bolt et al.; Pennington and FitzGerald) emphasized the importance of a comprehensive and valid biomarker assessment and a longitudinal characterization of large cohorts of PSO patients at increased risk for PsA transition. As a prerequisite to achieving a better understanding, it was emphasized that close collaboration between rheumatologists and dermatologists is the basis for successful research and patient care. Various genetic, epigenetic, cellular, and tissue markers, as well as clinical and imaging markers, alone or in combination, could serve as reliable prognostic tools for assessing the risk of developing PsA (Bolt et al.; Pennington and FitzGerald). These reviews showed that the research field is currently undergoing a rapid and successful development, with ever new findings and generation of important data.

As Pennington and Fitzgerald noted, prospective studies could validate the biomarker-based prognostic model in early and established disease stages, particularly to validate proteins that can distinguish PsA from PSO, to determine negative and positive predictive values, clinical utility, and cost-effectiveness (Pennington and FitzGerald). In addition, Bolt et al. have explicitly pointed out that prospective study designs should particularly focus on obtaining, in addition to liquid biopsies, serial tissue samples of skin, synovium, or entheseal tissue to reveal which molecular and cellular processes change during the transition from PSO to PsA. Many efforts are being undertaken in this field, particularly in clinical research, to unravel the precise immunopathogenesis of PsA. Fortunately, prospective clinical trials are currently being undertaken in this area, for example, on entheseal biopsies (EudraCT 2018-004734-15). Together with animal models that mimic this transition, these studies will contribute to a better understanding of PsA development and ultimately will lead to the development of treatments that prevent the onset of PsA. Ultimately, the mid-term goal is to design stratified clinical trials for primary PsA prevention and to test and make available to our patients a targeted and patient-centered therapy.

What we further learned from our Research Topic is that the use of digital health technologies (eHealth) could help to provide early and reliable disease prognosis and diagnosis while conserving health resources (Fagni et al.). Various eHealth solutions such as telemedicine, mobile technologies, and in particular symptom checkers, could be helpful in this regard. Telemedicine enables remote rheumatology visits and consultations, while mobile technologies can improve monitoring by allowing patients to continuously self-report symptoms and disease-related parameters. Symptom checkers have the potential to refer patients to a physician earlier in their disease, minimizing diagnostic delays. Overall, these interventions could lead to earlier diagnosis of arthritis, improved monitoring, and better control of the disease while increasing the capacity of referral centers. The review (Fagni et al.) emphasizes the great potential of these strategies, but also states that to date, promising results have been observed primarily in rheumatoid arthritis and less so in psoriatic disease. Therefore, until validated tools for PsA are developed and tested in clinical trials, the potential of eHealth in this area remains partly speculative. If successful, these new digital approaches could address diagnostic delays and low adherence, improve disease surveillance, and ultimately lead to better disease outcomes.

In the original paper presented in this Research Topic, Zabotti et al. demonstrated that it is worthwhile to distinguish between peripheral and axial forms of spondyloarthropathy, especially with regard to treatment patterns and clinical characteristics. It was shown that (i) peripheral SpA patients had significantly more skin and nail PSO than axial ones and (ii) that most axial SpA patients (77%) started a biological therapy while over half of peripheral patients started with conventional disease-modifying antirheumatic drugs. These data are important because they show once again that psoriatic disease plays an important role for the entire spectrum of spondyloarthropathies.

In summary, our Resarch Topic demonstrated that the field of early diagnosis of PsA is undergoing dramatic change, intensive research with productive results is emerging, and there is therefore reasonable hope that in the future a greater proportion of PSO patients will be prevented from transition to PsA. In the coming years, due to the concentrated global research efforts, for example in the Innovative Medicines Initiative HIPPOCRATES, important research results are expected and will contribute to enable patient care based on precision medicine.

## Author Contributions

All authors listed have made a substantial, direct and intellectual contribution to the work, and approved it for publication.

## Conflict of Interest

The authors declare that the research was conducted in the absence of any commercial or financial relationships that could be construed as a potential conflict of interest.

## Publisher's Note

All claims expressed in this article are solely those of the authors and do not necessarily represent those of their affiliated organizations, or those of the publisher, the editors and the reviewers. Any product that may be evaluated in this article, or claim that may be made by its manufacturer, is not guaranteed or endorsed by the publisher.
